# Intermittent and Mild Cold Stimulation Maintains Immune Function Stability through Increasing the Levels of Intestinal Barrier Genes of Broilers

**DOI:** 10.3390/ani13132138

**Published:** 2023-06-28

**Authors:** Lu Xing, Tingting Li, Yong Zhang, Jun Bao, Haidong Wei, Jianhong Li

**Affiliations:** 1 College of Life Science, Northeast Agricultural University, Harbin 150030, China; 2 College of Animal Science and Technology, Northeast Agricultural University, Harbin 150030, China; 3 Key Laboratory of Chicken Genetics and Breeding, Ministry of Agriculture and Rural Affairs, Harbin 150030, China

**Keywords:** broiler, duodenum, jejunum, production performance, intestinal barrier, immunoglobulin

## Abstract

**Simple Summary:**

Cold is an important limiting factor in the development of the livestock and poultry industry, which has adverse effects on the intestinal barrier and immune function of animals. While appropriate cold stimulation can improve immune function, it remains unclear whether intermittent and mild cold stimulation can improve production performance, intestinal barrier function and stress tolerance through improving immunoglobulins and intestinal barrier gene levels. In this study, broilers were subjected to intermittent and mild cold stimulation and acute cold stress to assess its effects on production performance, intestinal barrier and immune function. The results indicate that intermittent and mild cold stimulation can improve the production performance, intestinal barrier and acute cold stress resistance of broilers through modulating the levels of intestinal barrier genes and immunoglobulins. We hope to provide a theoretical basis for the development of husbandry in cold environments.

**Abstract:**

In order to improve the adaptability of broilers to low-temperature environments and their ability to resist acute cold stress (ACS), 240 one-day-old broilers were selected and randomly divided into three groups. The control treatment (CC) group was raised at the conventional feeding temperature from 1–43 days (d), the cold stimulation treatment (CS) group was kept at 3 °C below the temperature of CC at 1 d intervals for 3 and 6 h from 15 to 35 d, namely, CS3 and CS6, respectively. Then, all broilers were kept at 20 °C from 36 to 43 d. ACS was then carried out at 44 d, and the ambient temperature was dropped to 10 °C for 6 h. The study investigated the production performance, as well as levels of intestinal barrier genes (including *Claudin-1*, *E-cadherin*, *Occludin*, *ZO-1*, *ZO-2* and *Mucin2*), secretory IgA in duodenum and jejunum, and immunoglobulins (IgA and IgG) in serum. The results showed that IMCS could increase the daily weight gain and decrease the feed conversion ratio. During IMCS, the expression levels of intestinal barrier genes were up-regulated and the content of secretory IgA was increased. When IMCS ceased for one week, the level of immunoglobulins in serum stabilized, and the expression levels of *Occludin*, *ZO-2* and *Mucin2* still maintained high levels. After ACS, broilers that received IMCS training maintained high levels of intestinal barrier genes and secretory IgA.

## 1. Introduction

Animals living in cold regions often suffer from low temperature that can cause damage to various organs, leading to decreased production performance, compromised immune function and disrupted internal homeostasis [1,2,3]. The intestinal tract is a protective barrier; it plays an essential role in immunity [4,5]. The intestinal mucosal barrier as a protective barrier can prevent the loss of water and electrolytes and the invasion of microorganisms in the lumen [6,7]. The physical intestinal barrier is mainly composed of intestinal epithelial cells and tight junctions (TJs) [8]. TJs play a crucial role in intercellular connections [9,10]. The absence of a TJ complex can impair barrier protection function, affecting intestinal permeability, which can be used as an indicator to evaluate the integrity of intestinal mucosa [11]. Fanning and Krause et al. discovered that Claudins and ZO-1 proteins can regulate epithelial permeability and help maintain the integrity of the intestinal barrier [10,12]. The levels of *Claudin-1* and *Occludin* can be used as biomarkers to assess broilers’ intestinal barrier function [13,14]. Stress can inhibit the expression levels of TJ proteins, increase intestinal permeability, destroy the integrity of the intestine and result in intestinal barrier dysfunction in broilers [15,16,17]. For instance, heat stress (35 °C) can reduce levels of TJ proteins and increase the permeability of tight junctions between cells of broilers [18]. Zhao et al. found that low temperature could significantly shorten the villus height of the small intestine of broilers and seriously damage the intestine [19]. However, appropriate cold or hot stimulation can improve intestinal tightness, thereby enhancing intestinal barrier function. Broilers subjected to heat stress (38–39 °C) for 5 days could significantly increase the TJ protein levels in the jejunum [20]. Cold stimulation every other day (3 °C below the conventional temperature) could relieve intestinal damage when broilers are subjected to acute cold stress [21]. Therefore, the impact of the environment on organisms is twofold, and reasonable use of environmental stimuli can help maintain the integrity of the intestinal barrier.

Immunoglobulins are crucial effectors of the immune system, and levels of immunoglobulins can vary with changes in the internal and external environment of the body. When environmental changes are excessive, a reduction in the content of immunoglobulin in animal serum can lead to damage to the body. Heat stress (40 °C) for 4 weeks reduced the IgA and IgG content in serum and affected the development of immune function in broilers [22]. Appropriate cold stimulation can raise the content of immunoglobulin to a certain extent. Mild cold stimulation at 3 °C below the normal feeding temperature for broilers can up-regulate IgA and IgG expression levels in the spleen and activate the immune system [23]. After acute cold stress, the content of IgA in the ileum of broilers with cold training was significantly higher than that of broilers without cold training, aiding the body in resisting cold environments [21]. Therefore, the level of immunoglobulin can reflect the immune function of the body.

Low temperature is one of the important factors limiting the livestock and poultry industry in northern regions, as cold stress often causes damage to animals. However, reasonable utilization of cold environments can have positive effects. Some studies indicated that proper cold stimulation can enhance the intestinal immunity of broilers [21,23]. It remains unclear whether intermittent and mild cold stimulation (IMCS) can enhance the production performance and intestinal immune function of broilers through enhancing the integrity of the intestinal barrier and the levels of immunoglobulins. In the study, broilers were subjected to IMCS training at 3 °C below normal rearing temperature every other day, aiming to explore the effect of IMCS on production performance, the intestinal barrier and immunity, and the study tried to find out its potential molecular mechanism. We hope to discover a suitable cold stimulation training program to help the body adapt to low-temperature environments and provide a theoretical basis for the establishment of cold adaptation strategies for poultry production practices in cold regions.

## 2. Materials and Methods

### 2.1. Animals and Experimental Design

**Figure 1:**
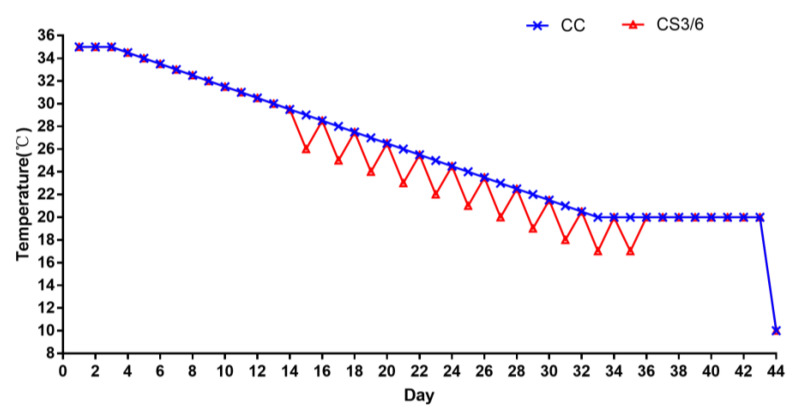
The specific experimental temperature scheme.

Two hundred and forty one-day-old Ross 308 broilers were subjected to three treatments randomly; each treatment had five replicates (16 broilers per replicate). All broilers were reared in battery cages located in climate-controlled rooms. The control group (CC) was raised at the standard feeding temperature, which was maintained at 35 °C for 1–3 days (d) then gradually reduced by 0.5 °C every day until it reached 20 °C on day 35. The feeding temperature of the cold stimulation group (CS) was the same as that of the CC group from 1 to 14 d, and the temperature at one-day intervals from 15–35 d was 3 °C below the CC group. The duration of cold stimulation was 3 h (CS3) and 6 h (CS6), respectively. From day 36 to 43, broilers in all three experimental groups were kept at a temperature of 20 °C. On day 44, all broilers in the three treatment groups were treated with acute cold stress (ACS) of 10 °C for 6 h. The duration of ACS was from 07:00 to 13:00. The specific feeding temperature is shown in Figure 1. The relative humidity of the room was kept at 60–70% during days 1–14 and 40–50% during days 15–44. Throughout the study, the birds were given free access to water and feed. The feed supply included a commercial starter diet for 1–3 weeks (metabolizable energy [ME] of 12.1 MJ/kg and crude protein [CP] of 21.0%) and a commercial growing-finishing diet for 4–6 weeks (ME of 12.6 MJ/kg and CP of 19.0%) (Baishicheng Animal Husbandry, Harbin, China). The main composition of the commercial diets included corn, soybean meal, vitamin A, vitamin D3, NaCl, copper sulfate, calcium chloride, choline, etc.

### 2.2. Sample Collection

In this study, four broilers in each group were randomly selected and euthanized for blood, duodenum and jejunum at 22, 36 and 43 d and after 6 h of ACS. The duodenum and jejunum were taken out, rapidly frozen and transferred to a −80 °C refrigerator. Blood samples were stewed and centrifuged at 2000× *g* at 4 °C for 15 min. Then, the supernatants were sucked from the centrifuge tube and stored at −20 °C.

### 2.3. Evaluation of Broiler Production Performance

Five representative broilers were grabbed from each group randomly, and weight gain was recorded on the first day of weeks 3, 4, 5 and 6. Feed addition and surplus of each cage were counted every day. The daily weight gain and daily feed intake were recorded and averaged. The number of dead broilers in each group was recorded and excluded from the test, and the average value was measured for the remaining broilers. The formula of the feed conversion ratio was daily feed intake/daily weight gain.

### 2.4. RNA Extraction and Reverse Transcription

According to the instructions, total RNA from the duodenum and jejunum was extracted using Rnaiso plus (Bao Bioengineering Co., Ltd., Dalian, China). RNA concentration and OD260/OD280 value were detected with an ultramicro spectrophotometer (IMPLEN, p330, Munich, Germany). RNA concentration was adjusted to 1 μg/μL and the OD value was within the range of 1.8–2.1. Complementary DNA (cDNA) was synthesized using a PrimeScript^®^ RT reagent Kit with gDNA Eraser (Perfect Real-Time) (TaKaRa, Shiga, Japan). The specific reaction system is listed in Table 1.

### 2.5. Quantitative Real-Time PCR (qRT-PCR) Analysis

Target genes (*Claudin-1*, *E-cadherin*, *Occludin*, *ZO-1*, *ZO-2*, *Mucin2*) and the reference gene (*β-actin*) were synthesized by Shanghai Sangong Bioengineering Co., Ltd. (Shanghai, China). The primer sequence is shown in Table 2. According to the manufacturer’s instructions, qRT-RCR was performed on AriaMx Real Time PCR instrument (Agilent, Santa Clara, CA, USA) using THUNDERBIRDSYBR qPCR Mix (Toyobo, Japan). The reaction procedure was as follows: 95 °C for 60 s, followed by 40 cycles of 95 °C for 15 s, and 60 °C for 1 min. The method of 2^−ΔΔ*C*t^ was used to calculate the relative mRNA levels of the target genes.

### 2.6. Western Blot Analysis

Western and IP cell lysates (Biosharp, Beijing, China) containing 1% PMSF (SparkJade, Harbin, China) were used to extract proteins from the duodenum and jejunum. A BCA protein detection kit (Biosharp, Beijing, China) was used to determine the protein concentration. The concentration was adjusted to 4 mg/mL. The methods of Western blotting were according to our previous reports [21]. Briefly, equal amounts of total protein (40 mg/condition) were separated using 10% SDS-PAGE gel (Beyotime, Shanghai, China) and transferred to nitrocellulose membrane with a semi-dry transfer instrument. The membrane was sealed in 5% skimmed milk for 2 h and then washed with 1× PBST three times. The nitrocellulose membranes were incubated with the primary antibodies against Claudin-1 (1:500, WanleiBio, Shenyang, China), E-cadherin (1:1000, Sangong, Shanghai, China), Occludin (1:1000, Sangong, Shanghai, China) and β-actin (1:9000, Zenbo, Chengdu, China) at 4 °C overnight. After cleaning the membrane with 1× PBST 3 times, an HRP goat anti-rabbit IgG (1:9000, ABclonal, Wuhan, China) was added and incubated with the membrane for 1 h. The protein bands were washed with 1× PBST three times, visualized using ECL chemiluminescence kit (Biosharp, Beijing, China) and scanned with a grayscale scanner (CLINX, Shanghai, China). Finally, Image J software V1.8.0 (NIH, Bethesda, MD, USA) was used for grayscale analysis. The gray value ratio of target protein to β-actin was used to express the relative expression levels of proteins.

### 2.7. Elisa Detection

The levels of immunoglobulin (IgA, IgG and IgM) in serum of broilers at 22, 36 and 43 d and secretory IgA (SIgA) in the duodenum and jejunum at day 22, 36, 43 and Y6 were detected via enzyme-linked immunosorbent assay (ELISA), according to the instructions provided by the manufacturer (mlbio, Shanghai, China).

### 2.8. Statistics and Analysis

All results were analyzed using IBM SPSS 21.0 software (IBM, Armonk, NY, USA). For all data, the normal distribution was tested using the Kolmogorov–Smirnov test. One-way analysis of variance (ANOVA) with Duncan’s multiple comparisons was used to analyze the effect of intermittent and mild cold stimulation (IMCS) on production performance, the mRNA and protein expression levels of barrier genes in the duodenum and jejunum and the content of immunoglobulin in serum, duodenum and jejunum. The mean ± standard deviation (SD) was used to present the results, and a significant difference was expressed as *p* < 0.05.

## 3. Results

### 3.1. Production Performance of Broilers

The effect of IMCS on production performance is shown in Table 3. In the CS3 group, the feed conversion ratio was significantly decreased compared to the CC and CS6 groups (*p* < 0.05). The CS3 group had significantly higher daily weight gain compared to the CC and CS6 groups (*p* < 0.05). The daily food intake between the CC and CS groups was not significantly different (*p* > 0.05).

### 3.2. mRNA Levels of Intestinal Barrier Genes in the Duodenum

**Figure 2:**
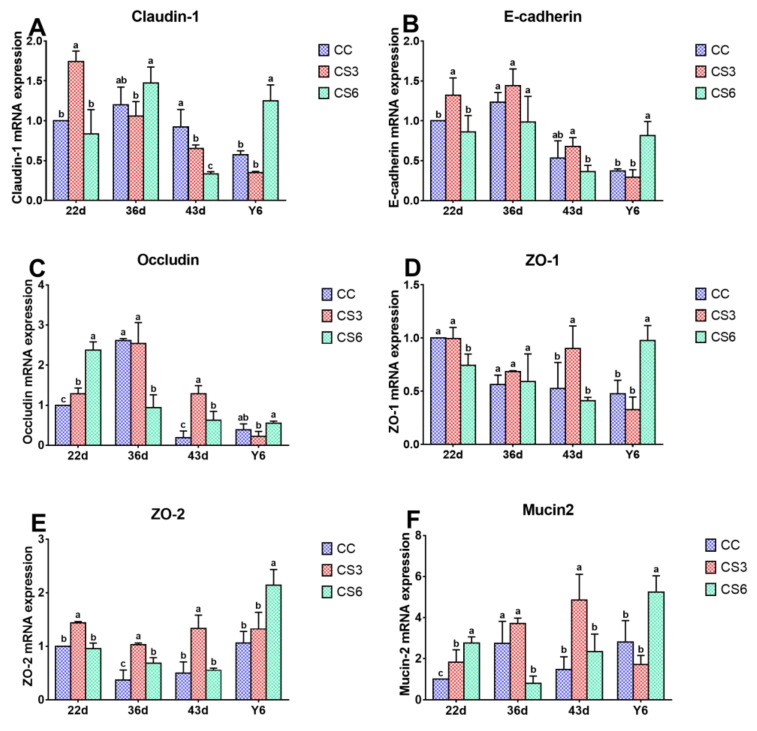
Effects of cold stimulation on mRNA levels of *Claudin-1* (**A**), *E-cadherin* (**B**), *Occludin* (**C**), *ZO-1* (**D**), *ZO-2* (**E**) and *Mucin2* (**F**) in duodenum. Values are means ± SD. Different letters indicate significant differences among the treatment (a, b, c) (*p* < 0.05). Respective *p* values among groups are: *Claudin-1* (22 d: 0.000; 36 d: 0.076; 43 d: 0.001; Y6: 0.002); *E-cadherin* (22 d: 0.013; 36 d: 0.133; 43 d: 0.043; Y6: 0.008); *Occludin* (22 d: 0.000; 36 d: 0.009; 43 d: 0.000; Y6: 0.058); *ZO-1* (22 d: 0.003; 36 d: 0.556; 43 d: 0.012; Y6: 0.005); *ZO-2* (22 d: 0.000; 36 d: 0.001; 43 d: 0.001; Y6: 0.007); *Mucin2* (22 d: 0.001; 36 d: 0.005; 43 d: 0.027; Y6: 0.004).

Figure 2 displays the results of mRNA expression levels of barrier genes in the duodenum. At 22 d, the mRNA expression levels of *Claudin-1*, *E-cadherin* and *ZO-2* in CS3 were significantly higher than those in CC and CS6 (*p* < 0.05), but there were no significant differences between CC and CS6 (*p* > 0.05). Compared to CC, the mRNA levels of *Occludin* and *Mucin2* in CS were increased significantly (*p* < 0.05), and the levels in CS6 were significantly higher than those in CS3 (*p* < 0.05). In the CC and CS3 groups, a lower *ZO-1* mRNA level was found when compared to CS6 treatment (*p* < 0.05), but difference between CC and CS3 was not significant (*p* > 0.05). At day 36, the mRNA levels of *Occludin* and *Mucin2* in CC and CS3 were significantly up-regulated compared to CS6 (*p* < 0.05). A significantly higher *ZO-2* mRNA level was obtained in CS compared to CC (*p* < 0.05), and that in CS3 was significantly higher than that in CS6 (*p* < 0.05). A significant increase in *Claudin-1* levels was found in CS6 compared with CS3 (*p* < 0.05). The expression levels of other genes were changed slightly (*p* > 0.05). At 43 d, the mRNA levels of *Occludin*, *ZO-1*, *ZO-2* and *Mucin2* in CS3 were significantly enhanced compared to those in CC and CS6 (*p* < 0.05), a significantly higher *Occludin* mRNA level in CS6 was observed compared to CC (*p* < 0.05), while no significant difference was observed in other genes (*p* > 0.05). *Claudin-1* mRNA levels in CS groups were significantly down-regulated than those in the CC group (*p* < 0.05). The level of *E-cadherin* in the CS3 group was significantly up-regulated compared with the CS6 group (*p* < 0.05). After 6 h of ACS (Y6), the levels of all genes, except for *Occludin* in CS6, were significantly increased compared to CC and CS3 (*p* < 0.05), while the difference between the CC and CS3 groups was not significant (*p* > 0.05). Moreover, the *Occludin* mRNA level in CS6 was noted to be significantly up-regulated compared to that in CS3 (*p* < 0.05).

### 3.3. Protein Levels of Intestinal Barrier Genes in the Duodenum

**Figure 3:**
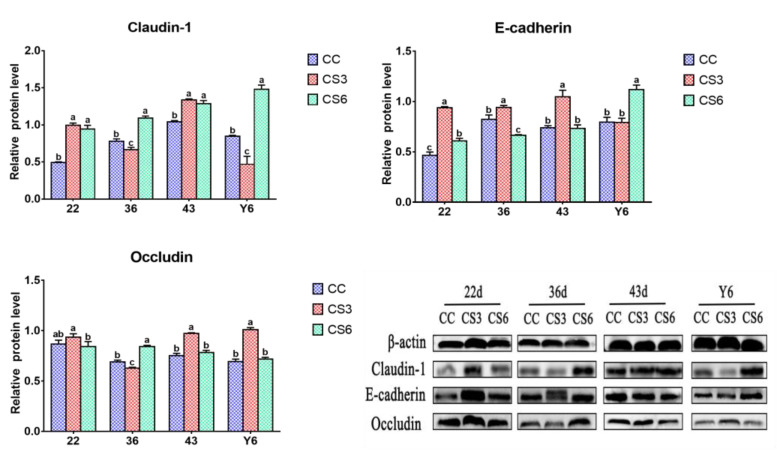
Effects of cold stimulation on protein levels of intestinal barrier genes in duodenum. Values are means ± SD. Different letters indicate significant differences among the treatment (a, b, c) (*p* < 0.05). Respective *p* values among groups are: Claudin-1 (22 d: 0.000; 36 d: 0.000; 43 d: 0.000; Y6: 0.000); E-cadherin (22 d: 0.000; 36 d: 0.000; 43 d: 0.000; Y6: 0.000); Occludin (22 d: 0.069; 36 d: 0.000; 43 d: 0.000; Y6: 0.000).

The results of protein expression levels of Claudin-1, Occludin and E-cadherin in the duodenum are shown in Figure 3. At 22 d, the protein expression level of Claudin-1 in CS was significantly increased compared to CC (*p* < 0.05); however, differences between the CS3 and CS6 groups was not significant (*p* > 0.05). The level of Occludin in CS3 was the highest among the three groups, but the difference was non-significant between CC and CS (*p* > 0.05). Compared to CC, a significant increase was presented in the protein level of E-cadherin in CS (*p* < 0.05), and a noticeably higher level was noted in E-cadherin in CS3 compared to CS6 (*p* < 0.05). At day 36, a significant increment in Claudin-1 and Occludin levels was found in CS6 when compared to CC and CS3 (*p* < 0.05), and those in CC were significantly higher than those in CS3 (*p* < 0.05). The E-cadherin level in CS3 was increased significantly compared to CC and CS6 (*p* < 0.05), and the level of E-cadherin in CC was significantly increased in comparison with CS6 (*p* < 0.05). At day 43, the CS groups had higher expression levels of Claudin-1 than the CC group (*p* < 0.05), but the difference between CS3 and CS6 was not significant (*p* > 0.05). The protein levels of Occludin and E-cadherin in CS3 were significantly higher than those in CC and CS6 (*p* < 0.05), while no significant difference was observed between the CC and CS groups (*p* > 0.05). After ACS for 6 h, the levels of Claudin-1 and E-cadherin were significantly increased compared to CC and CS3 (*p* < 0.05), and the Claudin-1 level in CC was significantly higher than that in CS3 (*p* < 0.05). The E-cadherin level had no significant difference between CC and CS3 (*p* > 0.05). A significant increase in Occludin levels was observed in CS3 when compared to CC and CS6 (*p* < 0.05), but there was no significant difference between CC and CS6 (*p* > 0.05).

### 3.4. mRNA Levels of Intestinal Barrier Genes in the Jejunum

**Figure 4:**
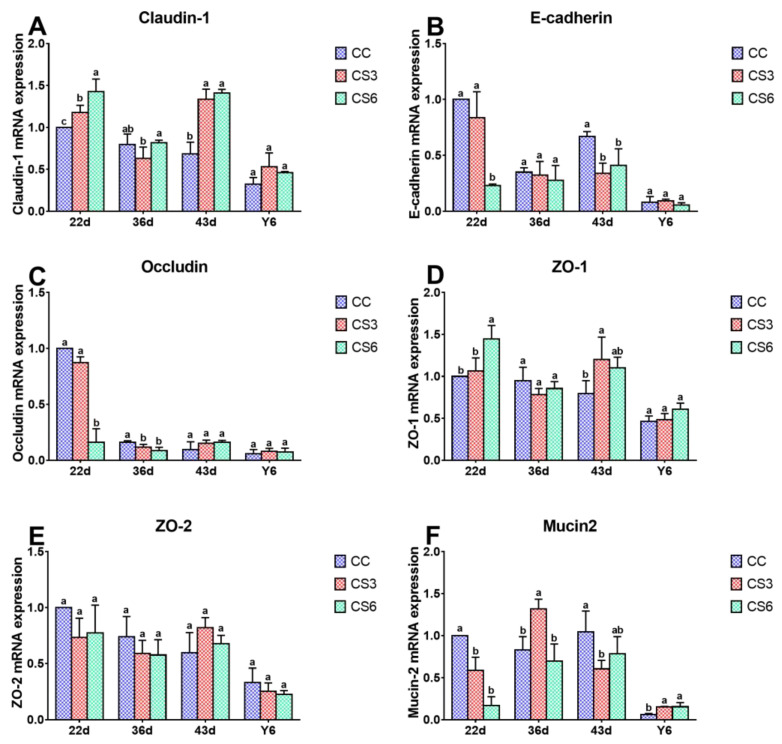
Effects of cold stimulation on mRNA levels of *Claudin-1* (**A**), *E-cadherin* (**B**), *Occludin* (**C**), *ZO-1* (**D**), *ZO-2* (**E**) and *Mucin2* (**F**) in jejunum. Values are means ± SD. Different letters indicate significant differences among the treatment (a, b, c) (*p* < 0.05). Respective *p* values among groups are: *Claudin-1* (22 d: 0.001; 36 d: 0.070; 43 d: 0.000; Y6: 0.282); *E-cadherin* (22 d: 0.002; 36 d: 0.639; 43 d: 0.034; Y6: 0.539); *Occludin* (22 d: 0.000; 36 d: 0.006; 43 d: 0.122; Y6: 0.759); *ZO-1* (22 d: 0.002; 36 d: 0.172; 43 d: 0.039; Y6: 0.188); *ZO-2* (22 d: 0.098; 36 d: 0.382; 43 d: 0.159; Y6: 0.453); *Mucin2* (22 d: 0.000; 36 d: 0.023; 43 d: 0.111; Y6: 0.032).

Figure 4 shows the effect of IMCS and ACS on mRNA levels of barrier genes in the jejunum. After seven-day IMCS (at 22 d), compared to CS groups, the level of *Mucin2* mRNA in CC was significantly increased (*p* < 0.05). Compared to CS6, significantly higher mRNA levels of *E-cadherin* and *Occludin* and significant lower levels of *Claudin-1* and *ZO-1* were found in CC and CS3 (*p* < 0.05), and *Claudin-1* in CS3 had a significantly higher level compared to the CC group (*p* < 0.05). However, no significant difference between the CC and CS3 groups was observed for *E-cadherin*, *Occludin* and *ZO-1* (*p* > 0.05). Among the CC and CS groups, the level of *ZO-2* was not significantly different (*p* > 0.05). After fourteen-day IMCS (36 d), in the CC group, the mRNA level of *Occludin* was significantly up-regulated compared to CS (*p* < 0.05), whereas there was no significant difference between the CS groups (*p* > 0.05). Compared with CC and CS6, *Mucin2* level in CS3 exhibited a marked increase (*p* < 0.05), but there was no significant difference between CC and CS6 (*p* > 0.05). *Claudin-1* mRNA level in CS6 was significantly increased compared to CC (*p* < 0.05). No significant difference was found for other genes (*p* > 0.05). At 43 d, compared with CC, the mRNA expression levels of *Claudin-1* in CS and *ZO-1* in CS3 were significantly up-regulated (*p* < 0.05), while *E-cadherin* in CS and *Mucin2* in CS3 were significantly down-regulated (*p* < 0.05). There was no significant difference in the levels of *Claudin-1* and *E-cadherin* between the CS3 and CS6 groups (*p* > 0.05). After ACS, the mRNA level of *Mucin2* in CS was significantly higher than that in CC (*p* < 0.05), but there was no significant difference in *Mucin2* between the CS groups (*p* > 0.05) and for other genes among the three groups (*p* > 0.05).

### 3.5. Protein Levels of Intestinal Barrier Genes in the Jejunum

**Figure 5:**
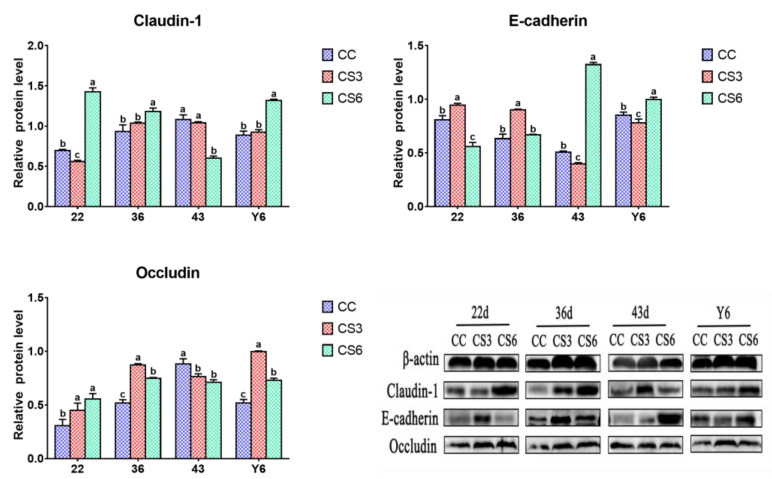
Effects of cold stimulation on protein levels of intestinal barrier genes in jejunum. Values are means ± SD. Different letters indicate significant differences among the treatment (a, b, c) (*p* < 0.05). Respective *p* values among groups are: Claudin-1 (22 d: 0.000; 36 d: 0.004; 43 d: 0.000; Y6: 0.000); E-cadherin (22 d: 0.000; 36 d: 0.000; 43 d: 0.000; Y6: 0.000); Occludin (22 d: 0.006; 36 d: 0.000; 43 d: 0.003; Y6: 0.000).

Figure 5 shows the effect of IMCS and ACS on protein levels of barrier genes in the jejunum. At 22 d, the level of Claudin-1 in CS6 was higher than in CC and CS3 (*p* < 0.05), and that in CC was significantly higher than that in CS3 (*p* < 0.05). Cold stimulation resulted in a significantly higher Occludin protein level in the CS groups compared to the CC group (*p* < 0.05). Compared to CC and CS6, a significant increase in the protein levels of E-cadherin was found in CS3 (*p* < 0.05), and the E-cadherin level in CC was significantly higher than that in CS6 (*p* < 0.05). At 36 d, the Claudin-1 level in CS6 was increased significantly compared to CC and CS3 (*p* < 0.05), and the difference was not significant between CC and CS3 (*p* > 0.05). The levels of Occludin and E-cadherin in CS3 were significantly higher than those in CC and CS6 (*p* < 0.05), and compared to CC, Occludin in CS6 had a significantly higher level (*p* < 0.05), but no significant difference was observed in E-cadherin between CC and CS6 (*p* > 0.05). At 43 d, compared to CS6, the Claudin-1 level in CC and CS3 was significantly up-regulated (*p* < 0.05). The level of Occludin in CC was significantly increased in comparison with CS (*p* < 0.05). CS6 had a significantly higher E-cadherin level compared to CC and CS3 (*p* < 0.05), and had a significantly higher level than CS3 (*p* < 0.05). After ACS for 6 h, compared to CC and CS3, the levels of Claudin-1 and E-cadherin in CS6 were significantly increased (*p* < 0.05), and the E-cadherin level in CC was up-regulated compared to CS3 (*p* < 0.05), but no significant difference was observed in Claudin-1 between CC and CS3 (*p* > 0.05). The level of Occludin in CS was significantly increased compared to CC (*p* < 0.05), and that in CS3 was significantly higher than that in CS6 (*p* < 0.05).

### 3.6. Levels of Immunoglobulin in Serum, Duodenum and Jejunum

**Figure 6:**
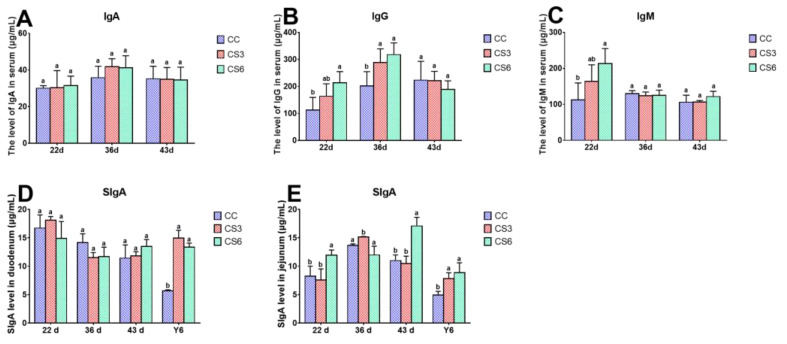
Effects of IMCS and ACS on the content of IgA (**A**), IgG (**B**) and IgM (**C**) in serum, SIgA (**D**,**E**) in duodenum and jejunum. Values are means ± SD. Different letters indicate significant differences among the treatment (a, b) (*p* < 0.05). Respective *p* values among groups are: IgA (22 d: 0.938; 36 d: 0.316; 43 d: 0.991); IgG (22 d: 0.035; 36 d: 0.022; 43 d: 0.560); IgM (22 d: 0.035; 36 d: 0.770; 43 d: 0.263); SIgA in duodenum (22 d: 0.367; 36 d: 0.106; 43 d: 0.304; Y6: 0.015); SIgA in jejunum (22 d: 0.167; 36 d: 0.019; 43 d: 0.105; Y6: 0.019).

Figure 6 shows the effect of IMCS and ACS on the content of immunoglobulin in serum (Figure 6A–C), duodenum and jejunum (Figure 6D,E). After seven-day IMCS (at 22 d), the contents of IgG and IgM in CS6 were significantly enhanced compared to CC in serum (*p* < 0.05), and those in CS3 were higher than those in CC, but the difference was not significant (*p* > 0.05). A significantly higher content of SIgA in CS6 than that in CC and CS3 was found in the jejunum (*p* < 0.05), but CC and CS3 had no significant difference (*p* > 0.05). No statistically significant difference in contents of IgA in serum and SIgA in the duodenum was observed among the three groups (*p* > 0.05). At 36 d, the CS groups had significantly higher IgG level compared to the CC group (*p* < 0.05). The content of SIgA in the jejunum in CC and CS6 was remarkably decreased compared to that in CS3 (*p* < 0.05). The contents of IgA and IgM in serum and SIgA in the duodenum had no significant difference among CC and CS groups (*p* > 0.05). At 43 d, the contents of immunoglobulin except for SIgA in the jejunum were not significantly different among the three groups (*p* > 0.05). The content of SIgA in the jejunum in CS6 was significantly higher than that in CC and CS3 (*p* < 0.05). After ACS for 6 h, the content of SIgA in the duodenum and jejunum in CS was significantly increased compared to CC (*p* < 0.05).

## 4. Discussion

Environmental temperature has a significant impact on broiler production performance, including egg production, food intake, weight gain and ketone body quality [3,24,25]. A low-temperature environment leads to significantly decreased body weight gain, pectoral weight and thigh muscle weight of broilers [26]. However, appropriate cold stimulation would prevent adverse effects on production performance. Blahova et al. found that after 20 days of cold stimulation (4–13 °C), no significant difference was found in the body weight gain of broilers in the control group and cold stress group [27]. Moreover, Shinder et al. reported that the survival rate of broilers significantly increased after exposure to repeated short-term cold training [28]. The above results were similar to ours. In the present study, the daily feed intake among CC and CS groups was similar, which may be due to the fact that cold stimulation (3 °C below feeding temperature of the CC group) was relatively mild. Broilers can adapt to the environment through exerting thermoregulation, without the need for increasing daily feed intake to increase heat production. The daily weight gain of broilers in the CS3 group was significantly higher than that of the CC and CS6 groups, but no significant difference was observed between the CC and CS6 groups; this could be partly explained by the fact that IMCS applied for 3 h increased daily weight gain through increasing fat accumulation, whereas IMCS applied for 6 h would eliminate the accumulation effect observed in broilers submitted to ICMS for 3 h. The feed conversion ratio of broilers in the CS3 group was the lowest, and the daily feed intake among all groups was similar, although the daily weight gain of broilers in the CS3 group increased. Collectively, the results showed that IMCS could increase the production performance of broilers.

TJ is the most important connection between intestinal cells. TJ only allows small molecules to pass through and hinders the passage of macromolecules and microorganisms, which plays an important role in maintaining the integrity of mucosa and the stability of barrier function [9,10]. In the present study, at the beginning of CS (22 d), the mRNA and protein levels of *Claudin-1* and *Occludin* in duodenum in CS3 and that of *Claudin-1* in the jejunum in CS6 were up-regulated to resist the influence of cold stimulation on intestinal mucosa. When the IMCS ended (36 d), the protein levels of Claudin-1 in CS6 in the duodenum and jejunum and that of Occludin in CS3 in the jejunum were increased. The results indicated that the 21-day IMCS training (3 °C below ambient temperature) could not cause damage to the intestinal mucosa and could promote the expression and reasonable distribution of intestinal TJ to a certain extent, thus maintaining the integrity of the mucosa and the stability of the intestinal barrier function. The protein level of Occludin in Caco-2 cells was increased after heat stress (39 or 41 °C) [29]. A study showed that cold stimulation at 16 °C for 72 h could enhance the level of *Occludin* in the jejunum of broilers [30], which is consistent with our results. ZO-1 is the key to the polymerization of Claudins [31]. In the study, one week after IMCS (43 d), the mRNA levels of *ZO-1* and *ZO-2* in the duodenum and jejunum of the CS3 group were increased, proving that IMCS could promote *Claudin* polymerization through increasing the expression levels of *ZO-1* and *ZO-2*, making the intestinal barrier tighter. Consistent with our results, Zhou et al. found that 72 h cold treatment resulted in increased ZO-1 levels. E-cadherin, which distributes epithelial cells and is involved in cell adhesion, is a calcium-dependent transmembrane protein [32]. Mucin2 is abundant in the intestinal tract of animals and plays an important role in resisting the invasion of intestinal microorganisms [33]. The study found that the *E-cadherin* and *Mucin2* levels in CS3 in the duodenum were higher than those in CC from 22 d to 43 d, indicating that appropriate cold stimulation could up-regulate *E-cadherin* and *Mucin2* levels, improve the integrity of intestinal epithelial cells and the tightness between cells, protect the intestine from susceptible bacteria and maintain intestinal barrier function. This is consistent with a previous study showing that changes in environmental temperature can affect the expression of TJ genes and thereby alter intestinal permeability and barrier function [34]. In the study, the expression trend of TJ genes in the duodenum and jejunum is slightly different, which may be because TJ proteins differentiate and restrict solute channels according to molecular size and change with different intestines [35]. Overall, the study suggested that appropriate cold stimulation could enhance intestinal barrier function through promoting TJ gene levels, which is beneficial to the health and production performance of broilers.

As an important effector molecule of the immune system, immunoglobulin plays an important role in the process of resisting cold stimulation [36,37]. In this study, at the beginning of IMCS, the levels of IgG and IgM in the CS group and SIgA in the jejunum in CS6 were increased, and the immunoglobulin levels between the CC and CS groups had no remarkable difference at 43 d. This finding suggests that at the beginning of cold stimulation, broilers could increase the secretion of immunoglobulin to improve the intestinal immune function and resist the cold environment. Therefore, when broilers adapted to the environment, the content of immunoglobulin returned to a normal level. Increased barrier tightness during cold stimulation may be one reason for the increase in immunoglobulin levels, as a tighter barrier prevents pathogens from invading the intestinal tract and improves immune function. The increase in immunoglobulin content in the serum of broilers and the improvement of immune function reported by Sang Oh Park when exposed to temperatures 8 °C higher than the control group is consistent with our findings [38]. Carr et al. revealed that levels of IgG and IgM were improved when mice were submitted to cold stress. Thaxton et al. found that cold exposure could accelerate the synthesis rate of IgG in chickens and enhance humoral immunity to a certain extent [39]. The above results match our results. At the same time, the level of immunoglobulin can also affect production performance [40], which is one of the reasons for the improvement in production performance after IMCS training.

Epithelial cells are arranged on the surface of mucosa. These cells establish a selective permeation barrier between the internal and external environment, responsible for digesting food and absorbing nutrients. The study showed that after ACS, the protein levels of Claudin-1 and Occludin and the mRNA levels of *ZO-1* and *ZO-2* in the CS6 group were significantly up-regulated, indicating that increasing the expression levels of TJ genes in broilers of the CS group can increase the barrier tightness during the process of ACS and maintain the stability of intestinal function. Yang et al. found that heat stress for 1 h could enhance the *Claudin-1* mRNA level of the jejunum in ducks; this matches with our results [18]. Liu et al. revealed that broilers which were subjected to 21 days of cold stimulation training could significantly up-regulate *Claudin-1* and *ZO-2* mRNA levels in the ileum after ACS [21]. It indicated that the cold tolerance of the body could be improved via IMCS in the early stage. In the present study, *E-cadherin* and *Mucin2* levels in the duodenum in CS6 and *Mucin2* levels in the jejunum in CS were up-regulated after ACS. SIgA plays an important role in intestinal immune response. Immunoglobulin levels can also affect intestinal barrier function [41]. In the present study, after ACS, the level of SIgA in CS was notably higher than that in CC. These results indicated that the expression level of mucin could be increased via IMCS training in the early stage, and *E-cadherin* and *Mucin2* could be rapidly up-regulated during ACS, activating the body’s protective mechanism, improving intestinal immune function, preventing microorganisms from invading the intestinal tract and maintaining the health of the intestine. This agrees with the previous research results of Liu et al., who reported that the expression level of *Mucin2* in the ileum of broilers which had undergone cold training in the early stage did not change significantly when subjected to ACS, and the mRNA level of *IgA* increased significantly [21]. In the present study, no statistical significance in the content of IgA in the serum was found, indicating that the body could maintain stability of immune function through immunoregulation. According to Varasteh et al., the level of *E-cadherin* in the jejunum and ileum of broilers significantly increased after heat stress (38–39 °C) [20], further supporting the positive effects of appropriate stimulation. In conclusion, appropriate cold stimulation training can improve the expression level of mucin to a certain extent, which can effectively block the invasion of pathogenic microorganisms, enhance immune capacity and, thus, improve the ability to resist ACS.

## 5. Conclusions

IMCS training had a positive effect on production performance, the function of the intestinal barrier and immunity through upregulating levels of intestinal barrier genes and SIgA. In the process of ACS, the levels of intestinal barrier genes and SIgA in the CS group could be rapidly increased to maintain the integrity of the intestinal barrier and the overall health of broilers.

## Figures and Tables

**Table 1:** Reverse transcriptional reaction system.

Reagent	Usage
5× gDNA Eraser Buffer	2.0 µL
gDNA Eraser	1.0 µL
Total RNA	1 µg
RNase Free dH_2_O up to	10.0 µL
42 °C Water-bath for 2 min	
5× PrimeScript Buffer 2	4.0 µL
RT Primer Mix	1.0 µL
PrimeScript RT Enzyme Mix I	1.0 µL
RNase Free dH_2_O	4.0 µL
37 °C Water-bath for 15 min, then 85 °C Water-bath for 5 s	

**Table 2:** Gene-special primer sequences used for the study.

Gene	Gene Reference Sequence	Primer Sequences (5′-3′)
*β-actin*	NM_205518.1	Forward: CACCACAGCCGAGAGAGAAAT
		Reverse: TGACCATCAGGGAGTTCATAGC
*Claudin-1*	NM_001013611.2	Forward: TGGAGGATGACCAGGTGAAGA
		Reverse: CGAGCCACTCTGTTGCCATA
*E-cadherin*	NM 001039258.2	Forward: GACAGGGACATGAGGCAGAA
		Reverse: GCCGTGACAATGCCATTCTC
*Occludin*	NM 205128.1	Forward: TCATCGCCTCCATCGTCTAC
		Reverse: TCTTACTGCGCGTCTTCTGG
*ZO-1*	XM 413773.4	Forward: TGTAGCCACAGCAAGAGGTG
		Reverse: CTGGAATGGCTCCTTGTGGT
*ZO-2*	XM_025144669.1	Forward: CGGCAGCTATCAGACCACTC
		Reverse: CACAGACCAGCAAGCCTACAG
*Mucin2*	XM_421035	Forward: CAGCACCAACTTCTCAGTTC
		Reverse: TCTGCAGCCACACATTCTTT

**Table 3:** Effect of IMCS on production performance of broilers.

	CC	CS3	CS6	*p* Value
Feed conversion ratio	2.01 ± 0.07 ^a^	1.84 ± 0.06 ^b^	1.98 ± 0.07 ^a^	0.000
Daily feed intake (kg)	1.21 ± 0.37 ^a^	1.21 ± 0.36 ^a^	1.24 ± 0.40 ^a^	0.163
Daily weight gain (kg)	0.60 ± 0.14 ^b^	0.65 ± 0.15 ^a^	0.62 ± 0.14 ^b^	0.008

Values are means ± SD. Values with different lowercase letters are significantly different (*p* < 0.05).

## Data Availability

The data presented in this study are available upon request from the corresponding author.
